# Habitat assessment of Marco Polo sheep (*Ovis ammon polii)* in Eastern Tajikistan: Modeling the effects of climate change

**DOI:** 10.1002/ece3.4103

**Published:** 2018-04-24

**Authors:** Eric Ariel L. Salas, Raul Valdez, Stefan Michel, Kenneth G. Boykin

**Affiliations:** ^1^ Agricultural Research and Development Program College of Science and Engineering Central State University Wilberforce Ohio; ^2^ Department of Fish, Wildlife and Conservation Ecology New Mexico State University Las Cruces New Mexico; ^3^ IUCN Species Survival Commission Caprinae Specialist Group Kannawurf Germany

**Keywords:** ensemble forecasting models, global climate change, Marco Polo argali, Pamir Mountains, species distribution modeling, ungulates

## Abstract

Identifying the factors predicting the high‐elevation suitable habitats of Central Asian argali wild sheep and how these suitable habitats are affected by the changing climate regimes could help address conservation and management efforts and identify future critical habitat for the species in eastern Tajikistan. This study used environmental niche models (ENMs) to map and compare potential present and future distributions of suitable environmental conditions for Marco Polo argali. Argali occurrence points were collected during field surveys conducted from 2009 to 2016. Our models showed that terrain ruggedness and annual mean temperature had strong correlations on argali distribution. We then used two greenhouse gas concentration trajectories (RCP 4.5 and RCP 8.5) for two future time periods (2050 and 2070) to model the impacts of climate change on Marco Polo argali habitat. Results indicated a decline of suitable habitat with majority of losses observed at lower elevations (3,300–4,300 m). Models that considered all variables (climatic and nonclimatic) predicted losses of present suitable areas of 60.6% (6,928 km^2^) and 63.2% (7,219 km^2^) by 2050 and 2070, respectively. Results also showed averaged habitat gains of 46.2% (6,106 km^2^) at much higher elevations (4,500–6,900 m) and that elevational shifts of habitat use could occur in the future. Our results could provide information for conservation planning for this near threatened species in the region.

## 1 INTRODUCTION

One of the threats for large mammals in mountainous regions is climate change (Granados & Brodie, 2016), because mountain regions are sensitive to warming (IPCC 2007). It affects animal species by modifying their local habitats (Sexton, McIntyre, Angert, & Rice, 2009; IPCC (Intergovernmental Panel on Climate Change), 2007). Climate change has already altered the future availability of suitable habitats for mountain ungulates such as the wild yak (*Bos mutus*), chiru (*Pantholops hodgsonii*), kiang (*Equus kiang*), Tibetan gazelle (*Procapra picticaudata*), Przewalski's gazelle (*Procapra przewalskii*) (Luo, Jiang, & Tang, 2015; Schaller, 1998), and Himalayan musk deer (*Moschus chrysogaster*) (Lamsal, Kumar, Aryal, & Atreya, 2018). Warmer winters have been shown to increase adult survival of Alpine ibex (*Capra ibex*) (Jacobson, Provenzale, von Hardenberg, Bassano, & Festa‐Bianchet, 2004). Plant phenology changes due to warming caused reduced periods of access to high‐quality forage leading to declines in young recruitment of Alpine ibex and bighorn sheep (*Ovis Canadensis*) (Pettorelli, Pelletier, von Hardenberg, Festa‐Bianchet, & Coté, 2007). Habitats for the giant pandas on mountain ranges at the edge of the Tibetan Plateau have already shown fragmentation due to climate change (Shen et al., 2015). Also, climate change can decrease the abundance of species (Aryal, Brunton, & Raubenheimer, 2014; Thomas, 2004), not only because of disappearing habitats (Tamburello, Côté, & Dulvy, 2015), but also because mountain ungulates and other mammals are especially sensitive to warming temperatures in montane regions (Hansen, 2009; Haynes, Kung, Brandt, Yongping, & Waller, 2014). The harmful effects of climate change for ungulates are of critical importance as large mammals have a lower tolerance for deviation of body core temperature (van Beest & Milner, 2013; Fuller, Mitchell, Maloney, & Hetem, 2016; Jessen, 2001). Ungulates in southeastern Norway showed suboptimal movement behavior above their critical temperature thresholds in changing climates (van Beest & Milner, 2013). Therefore, it is essential to have a better understanding of how species distributions and habitat use patterns are affected by changing climate regimes in order to inform discussions on potential management options.

Argalis (*Ovis ammon*) are wild sheep restricted to Asia in Afghanistan, China, Kazakhstan, Kyrgyzstan, Mongolia, Pakistan, Russia, Tajikistan, and Uzbekistan (Valdez & Weinberg, 2011). Marco Polo argali (*O. a. polii*) occurs in eastern Tajikistan and adjacent areas of surrounding countries: China, Afghanistan, Pakistan, and Kyrgyzstan. They are highly desired big‐game trophies because of their long horns of up to 191 cm (75 in). Argalis are listed as endangered by the U.S. Fish and Wildlife Service throughout their range, except in Kyrgyzstan, Tajikistan, and Mongolia where they are designated as threatened. They are listed in CITES Appendix II and as Near Threatened in the IUCN Red List. Argali have declined in numbers and distribution during the last century (Harris & Reading, 2008; Valdez & Weinberg, 2011). Eastern Tajikistan has greater numbers of argali than any other country with a minimum of 24,000 in the Pamir region (Michel & Muratov, 2010; Valdez, Michel, Subbotin, & Klich, 2016). Due to the high elevation of the Pamirs, the region is extremely susceptible to climate‐driven threats, such as glacier recession (Khromova, Osipova, Tsvetkov, Dyurgerov, & Barry, 2006), and decrease in water storage and supply (Finaev, Liu, Bao, & Li, 2016) that could harmfully impact wildlife habitats (BIOFOR 2001). Glaciers play a crucial role in the hydrological cycle of high‐altitude regions (Nogués‐Bravo, Araujo, Errea, & Martinez‐Rica, 2007) as they, together with snow packs, provide freshwater and soil moisture necessary for the survival of vegetation communities. Because argali sheep sightings occur nearest to water sources where available forage is abundant (Salas, Boykin, & Valdez, 2016), and riparian habitats have been shown to be the strongest predictors for argali occurrence in Tibet (Singh, Yoccoz, Bhatnagar, & Fox, 2009) and in the Pamirs of Tajikistan (Salas, Valdez, & Michel, 2017), increased temperatures in mountainous regions could decrease forage availability in proximity to riparian areas and hence the amount of suitable habitat for argali. IPCC (Intergovernmental Panel on Climate Change) (2007) reported that retreat of various large glaciers could accelerate through the 21st century, which would initially increase summer flow but, in the long term, could reduce water availability in regions supplied by meltwater from major mountain ranges in Central Asia. This increased water scarcity could cause extensive habitat loss and degradation of mountain ecosystems (Breu & Hurni, 2003).

Hole et al. (2009) highlighted the need to project future habitat for species affected by climate change in order to aid in conservation and management efforts and identify critical habitats. Environmental niche models (ENMs) are valuable spatial ecological tools to better assess the relationship between species distributions and environmental factors, and understand future steps for species management and policy (Elith & Leathwick, 2009; Salas, Seamster, Boykin, Harings, & Dixon, 2017; Wiens, Seavy, & Jongsomjit, 2011). In this study, we used niche models (Elith & Leathwick, 2009; Peterson et al., 2011) to project availability of suitable environmental conditions for argali species using various climate projections derived from general circulation models (GCMs), and postprocessed via application of a simple statistical downscaling method. We contrasted future projected climate envelope suitability results produced from combinations of four GCMs and two greenhouse gas concentration trajectories for two future time periods. Our methodology used five niche models to predict environmental argali habitat suitability in contrast to all other wild sheep habitat climate change studies which used a single modeling technique. To account for the uncertainties of each statistical model and improve predictions of the current distribution of a species (Araújo, Whittaker, Ladle, & Erhard, 2005; Marmion, Parviainen, Luoto, Heikkinen, & Thuiller, 2009), we did an ensemble of five ENMs to assess the agreement or disagreement of their predictions. Combining ENMs within an ensemble could add information not shown in one single algorithm (Meller et al., 2014).

This is the first modeling study in Tajikistan to assess the impacts of climate change on argali habitat and the first quantitative study of factors driving climatic distribution patterns of argali. This study is directed toward filling this knowledge gap by combining statistical models with field sightings of argali collected in eastern Tajikistan. Our objective is threefold: to develop models of present‐day and potential future distributions of suitable environmental conditions for argali in mountainous regions in eastern Tajikistan, to compare how climate change impacts the future availability of habitat for argali, and to provide information of the diverse climatic and nonclimatic variables that could potentially affect argali habitat. Lastly, we hypothesized that there would be elevational habitat shifting of argali under future climate conditions when projected to years 2050 and 2070.

## 2 MATERIALS AND METHODS

The methodology was composed of three major steps: (i) processing of argali occurrence dataset and environmental variables, (ii) modeling of current environmental conditions that includes selection of ENMs and evaluation of current conditions, and (iii) modeling of future environmental conditions that includes selection of GCMs, representative concentration pathways (RCPs), and projection of current conditions to future conditions. We modeled argali habitat with two approaches, first using a model consisting of climatic and nonclimatic variables (all environmental variables), and a second model with only climatic variables.

### 2.1 Study area

**Figure 1:**
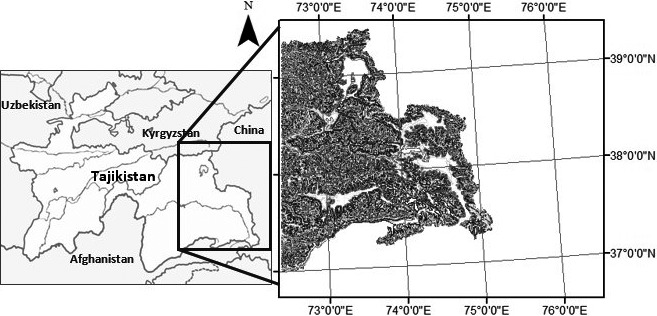
Location of study area in the eastern region of Tajikistan (inset) between latitudes 37°N to 40°N and longitudes 72°E to 76°E, and covers an area of approximately 41,000 km^2^. The Central Asian country of Tajikistan is bordered by Afghanistan, China, Kyrgyzstan, and Uzbekistan

The study area (Figure 1) is located in the eastern region of Tajikistan between latitudes 37°N to 40°N and longitudes 72°E to 76°E in the province of Gorno‐Badakhshan, and comprises approximately 41,000 km^2^. About 93% of Tajikistan is mountainous, and over half of the country is situated above 3,000 m. The terrains in the east of the country form the highest mountain systems in Central Asia, the Pamirs. The Pamir region is known as the “roof of the world” with wide and grassy valley floors, and meandering rivers and streams (Breu & Hurni, 2003). In the study area, elevations in the northern region range from 4,500 to 7,300 m, while those in the southern region range from 3,300 to 5,200 m above mean sea level (Salas, Valdez, & Boykin, 2015). The January mean temperature in the eastern Pamirs varies from −15 to −20°C with extreme seasonal temperature variations (WHC 2013). At lower elevations, the average January temperature is −1 to 3°C (NCCA 2003). Because of the specific climate conditions and varied landscape in Tajikistan, the country is deemed the main glacial center of Central Asia. The Fedchenko Glacier (907 km^2^), the largest glacier in Central Asia, is situated in the northern Pamir region of Tajikistan (Merzlyakova, 2002). In fact, the Tajik Pamirs alone provide approximately 60% of the freshwater reserves of Central Asian lowlands (Badenkov, 1992).

The dominant plant species are semishrubs such as teresken and sagebrush (*Artemisia* spp.); grasses (e.g., *Poa* spp.*, Festuca* spp.*, Hordeum* spp.*, Elymus* spp.); sedges (*Carex* spp. and *Kobresia* spp.); and forbs (e.g., *Dracocephalum* spp., *Oxytropis* spp., *Astragalus* spp., *Acantholimon* spp., *Crepis flexuosa*, and *Potentilla pamirica*) (Valdez et al., 2016).

The highest elevations of the northern region of the study area are in the Tajik National Park. The park is one of the largest high mountain protected areas in the Palearctic realm (Haslinger, Breu, Hurni, & Maselli, 2007). The state agency of Natural Protected Areas supervises all management activities in the park (WHC 2013). However, even in the Core Zone activities such as fuel wood cutting, livestock grazing, and hunting tourism take place (Weaver 2013), including a sport hunting concession primarily for Marco Polo sheep (Valdez et al., 2016).

The only other wild ungulate species in the study area is the Siberian ibex (*Capra sibirica*). Wild predators include wolf (*Canis lupus*), red fox (*Vulpes vulpes*), brown bear (*Ursus arctos isabellinus*), and snow leopard (*Panthera uncia*). There is limited mining activity at the southern edges of the park, and only one paved road with little traffic, and some unpaved roads in the study area. A barbed wire fence forms a barrier to argali movements with few gaps along the Tajik–Chinese border. Most of the area becomes inaccessible in the winter because of heavy snow accumulation. The majority of the study area outside of the park is assigned to private businesses as hunting concessions. Some of the hunting concession areas are patrolled to minimize illegal hunting, while other areas are unprotected (Valdez et al., 2016). Domestic ungulates include sheep (*Ovis aries*), goat (*Capra hircus*), yak (*Bos grunniens*), and cattle (*Bos Taurus*); domestic sheep are the most numerous followed by yak and few cattle and goats. Domestic animals, except for yak and few herds of sheep and goats, are moved to lower pastures during the fall, winter, and early spring (October–May) because of the harsh winter conditions at higher elevations.

### 2.2 Argali data

Argali occurrences were based on observations from field surveys over multiple years. Using the GPS location of the observers, the distance (measured by range finder or roughly guessed) and azimuth (measured with electromagnetic compass) between observers and observed animals, sightings of argali herds were georeferenced (Michel & Muratov, 2010; Valdez et al., 2016). Field surveys were conducted in March 2015, July 2015, August 2010, 2011, 2016, September 2013, and December 2009, 2013, 2014. Because clustered point occurrences could introduce potential spatial bias, our algorithm removed multiple presence localities in a grid and analyzed a single occurrence per pixel (Salas, Seamster, et al., 2017). A total 917 of 976 locations were used in the model. Further, we divided all occurrences into 70%–30% training–testing subsets when running our models.

### 2.3 Environmental variables

To build the models, we used 26 environmental variables (Table 1), of which 19 were bioclimatic variables from WorldClim datasets (Hijmans, Cameron, Parra, Jones, & Jarvis, 2005), and the rest were habitat and topographic variables that were previously identified as preferable for argali habitat (Salas, Valdez, et al., 2017). WorldClim provides climate projections statistically downscaled using a “delta method” approach to a spatial resolution of 30 arc‐sec, roughly 900 m at the equator. All raster images were resampled to the spatial resolution of the WorldClim data. We derived the green vegetation cover and sparse vegetation cover from the Global Land Cover 2000 project (Tateishi, Zhu, & Sato, 2000) and used them to represent forage abundance. Previous studies considered vegetation distribution as an important predictor in modeling the argali habitat (Salas et al., 2015; Singh et al., 2009). For escape terrain, we created a continuous distance around polygon patches with slopes ≥ 30° (Smith, Flinders, & Winn, 1991; Turner, Douglas, Hallum, Krausman, & Ramey, 2004). The processed DEM with a 1 arc‐second, or about 30 m (98 feet) resolution was sourced from NASA's Shuttle Radar Topography Mission (SRTM) digital elevation dataset that is available for download online (USGS 2016). We computed the slope and the aspect from the DEM. To further capture the relief characteristics of the landscape terrain, the terrain ruggedness index (TRI) was calculated from the DEM based on Sappington, Longshore, and Thomson (2007). Turner et al. (2004) showed that TRI could be a better predictor than proximity to escape terrain when both are used in the same modeling set. The TRI shows the average change in elevation between a center pixel and its eight neighboring pixels in a 3‐by‐3 window. All candidate variables were clipped to the extent of the study area.

**Table 1:** List of variables used in the model development for argali populations in eastern Tajikistan. Climate variable names and descriptions are based on WorldClim (Hijmans et al., 2005). An asterisk (*) denotes final variables selected by our algorithms in modeling the argali habitat

Predictor	Description	Unit
Climate		
Bioclim 1*	Annual mean temperature	°C
Bioclim 2*	Mean diurnal range	°C
Bioclim 3*	Isothermality	°C
Bioclim 4*	Temperature seasonality	°C
Bioclim 5	Maximum temperature of the warmest month	°C
Bioclim 6	Minimum temperature of the coldest month	°C
Bioclim 7	Temperature annual range	°C
Bioclim 8	Mean temperature of wettest quarter	°C
Bioclim 9*	Mean temperature of driest quarter	°C
Bioclim 10	Mean temperature of warmest quarter	°C
Bioclim 11	Mean temperature of coldest quarter	°C
Bioclim 12	Annual precipitation	°C
Bioclim 13	Precipitation of wettest month	°C
Bioclim 14	Precipitation of driest month	°C
Bioclim 15*	Precipitation seasonality	°C
Bioclim 16	Precipitation of wettest quarter	°C
Bioclim 17	Precipitation of driest quarter	°C
Bioclim 18*	Precipitation of warmest quarter	°C
Bioclim 19	Precipitation of coldest quarter	°C
Topographic Feature		
DEM	Digital elevation model	m
TRI (terrain ruggedness)*	Captured the difficulty of the landscape terrain $\sqrt{\text{Abs}({\left(\%\text{Max}\right)}^{2}-{\left(\%\text{Min}\right)}^{2})}$	
Aspect*	Derived from DEM	
Slope*	Derived from DEM	%
Vegetation cover*	Vegetation cover derived from the Global Land Cover 2000 project	
Sparse cover*	Sparse vegetation cover derived from the Global Land Cover 2000 project	
Distance to escape terrain*	Provides continuous distance from a defined slope of ≥30°	m
Distance to riparian areas*	Provides continuous distance from identified riparian areas	m

### 2.4 Species distribution modeling

To model the potential distribution of argali, we used the following statistical methods: generalized linear model (GLM), Random Forest (RF) (Breiman, 2001; Liaw & Wiener, 2002), boosted regression tree (BRT) (Elith, Leathwick, & Hastie, 2008), Maxent (Phillips, Anderson, & Schapire, 2006; Phillips & Dudík, 2008), and multivariate adaptive regression splines (MARS) (Leathwick, Elith, & Hastie, 2006). We selected ENMs based on their performance with presence‐only data (Elith et al., 2006). GLM, MARS, and BRT could be used for count data under the assumption that a count response could be modeled as Poisson (Talbert, 2012). The GLM is a linear regression adapted to binary count data. The method uses stepwise procedure to select covariates in the model. The MARS nonparametric algorithm builds flexible models by fitting piecewise logistic regressions. Although it has similarities with GLM, MARS is better in accommodating nonlinear responses to predictors and at the same time lessens the effects of outlying observations. The model RF uses decision trees through random grouping of the covariates. Random Forest models both interactions of the variables and their nonlinear relationships, and does not split the data into training and testing as RF utilizes bootstrapping to fit individual trees (Breiman, 2001). Like the Random Forest, BRT also uses decision trees, but the method is robust to missing observations. BRT uses cross‐validation by choosing models based on model comparisons of evaluation metrics (Elith et al., 2008). Maxent is best for presence‐only modeling. While observed absence is valuable in modeling, data are often not available and hence using only presence data are unavoidable (Talbert, 2012). Models were implemented in the modeling tool Software for Assisted Habitat Modeling (SAHM) run within VisTrails (Morisette et al., 2013; Talbert, 2012). The tool creates a workflow of the selected ENMs and develops models for present‐day conditions. As species lacked absence data, the tool randomly generated background points (i.e., pseudo‐absences [Phillips & Dudík, 2008]) within a 95% minimum convex polygon defined by the presence data.

We removed one of each pair of highly correlated (*r* > .7) (Dormann et al., 2013) environmental variables to avoid collinearity among predictors (Gama, Crespo, Dolbeth, & Anastácio, 2015). We made the choices between variables based on the results of a species‐specific literature search. In particular, we selected variables that were identified in one or more studies regarding the argali as having an effect on the argali's range or population dynamics. In cases where the results of the literature search could not differentiate between two highly correlated climatic variables, we used a qualitative assessment of the distribution of values of the variable at all presence points and of the relationship between the variable and species presence or pseudo‐absence (Talbert, 2012). We provided an asterisk in Table 1 to denote the final variables that were selected by our algorithms when modeling the argali habitat.

We produced ensemble maps for the current distributions. The ensemble map is a summation of binary maps generated from probability surfaces from each statistical modeling algorithm (Liu, Berry, Dawson, & Pearson, 2005; Lobo, Jiménez‐Valverde, & Real, 2008; Stohlgren et al., 2010). We optimized the threshold using specificity = sensitivity in discretizing the probability maps (Manel, Williams, & Ormerod, 2001). The final maps consisted of pixel values that represented the number of models in agreement to indicate that a particular pixel is suitable for argali. A pixel with a value of zero meant that none of the models identified bioclimatic suitability for the species at that location, while a value of 5 meant there was agreement across all five models. We used the current distributions estimated by the ensemble ENMs and projected each to the future.

### 2.5 Model evaluation

We evaluated confidence in individual model results in terms of concordance among the different distribution models. We had higher confidence that environmental conditions were suitable for a species when three or more (at least 60% of) algorithms were in agreement (e.g., Rehfeldt, Crookston, Sáenz‐Romero, & Campbell, 2012). We compiled information on various measures of model performance, including the area under the receiver operating characteristic (ROC) curve (AUC) for the test data, correct classification rate (Co%) (Fielding & Bell, 1997; Warren & Seifert, 2011), and the true skill statistic (TSS) (Allouche, Tsoar, & Kadmon, 2006) for each algorithm. The AUC value is the probability that the model would rank a randomly chosen presence observation higher than the randomly chosen absence observation. Swets (1988) classified values of AUC as follows: those >0.9 indicated high accuracy (excellent), from 0.7 to 0.9 indicated good accuracy, and those <0.7 indicated low accuracy. The TSS, a threshold‐dependent statistics, is presented as an improved measure of model accuracy that, unlike the common kappa statistics (Allouche et al., 2006; Cohen, 1960), is not dependent on species prevalence (i.e., proportion of occurrence points for which the species is present). Acceptable models are those with at least a TSS of 0.5, and excellent those with TSS around 0.7. We checked other qualitative assessments of model performance, which included the inspection of calibration and deviance of residual plots. Calibration plots indicate whether models tend to over or underpredict habitat suitability. Deviance of residual plots is used to identify individual data points that may require further inspection or whether there may be an important environmental layer missing from the model inputs (Morisette et al., 2013).

### 2.6 Species habitat forecasting

From WorldClim, we derived the eight sets of downscaled future climate projections produced by four GCMs highlighted in the National Climate Change Action (NCCA 2003) plan for Tajikistan. The selected GCMs include the following: Community Climate System Model version 4 (CCSM4; Gent et al., 2011), Hadley Centre Global Environment Model version 2‐Earth System (HadGEM2‐ES; Collins et al., 2011), Model for Interdisciplinary Research on Climate version 5 (MIROC5; Watanabe et al., 2010), and the Geophysical Fluid Dynamics Laboratory Coupled Model (GFDL‐CM3; Donner et al., 2011). We downloaded raster data for two RCPs (4.5 and 8.5) available for all selected GCMs and for two time periods (year 2050—average for 2041 to 2060 and year 2070—average for 2061 to 2080). RCP 4.5 was selected as it is more or less stable throughout the century among all RCPs in terms of reductions in greenhouse gas emissions (Arora et al., 2011; Roeckner, Giorgetta, Crueger, Esch, & Pongratz, 2011). For RCP 8.5, it is the most extreme scenario in that it entails the highest projected increase in the concentration of multiple greenhouse gases in the atmosphere (Vuuren, Edmonds, & Kainuma, 2011) and associated increases in global surface temperatures (Knutti & Sedláček, 2013). Results of the current argali distributions were projected to the future using the data from the four GCMs and according to the two RCPs. To avoid generating hundreds of map results, again we used ensembles to produce combination maps for the future distribution of the species. Each RCP result from the four GCMs was combined. In the end, a set of projection maps for the year 2050 and another set for the year 2070 according to each RCP were generated.

Finally, after considering the agreement (overlap) of at least three species distribution models and two GCMs, we compared the current and future ensemble maps to determine areas of stability, gains, and losses in suitable habitat conditions between present day and the two projected years. Suitable habitat is considered stable when present and future ensemble maps agree that the area is suitable for Marco Polo argali. There is gain in suitable habitat when future ensemble projects newly suitable areas beyond the extent of the present suitable map. There is loss of suitable habitat when present ensemble is converted to unsuitable condition in the future.

## 3 RESULTS

### 3.1 Model performance and variable importance

The AUC values for all models were above 0.80 (Table 2). Statistics for models with climate variables only are enclosed in parenthesis. BRT was the strongest with AUC = 0.94 (AUC = 0.93) followed by RF with 0.89 (0.86), Maxent with 0.86 (0.84), GLM with 0.84 (0.81), and MARS with 0.82 (0.81). BRT also had the highest values for TSS (0.73 and 0.70) and %Co (86.4 and 84.4). Results on the variable importance for models with both climatic and nonclimatic variables showed that the annual mean temperature (bioclim 1), precipitation of warmest quarter (bioclim 18), and temperature seasonality (bioclim 4) were identified as the three most relevant climatic variables for predicting habitat suitability and distribution of argali in all five models. The terrain ruggedness was also considered an important predictor, placing it in the top five among variable inputs. The TRI was the highest‐ranked nonclimatic variable. Other nonclimatic variables of importance included the vegetation cover, proximity to riparian areas, and aspect.

**Table 2:** Statistical results for the five different modeling algorithms. Model abbreviations are as follows: GLM, generalized linear model; MARS, multivariate adaptive regression splines; BRT, boosted regression tree, and RF, Random Forest. Statistics for models with climate variables are enclosed in parenthesis

Stats	GLM	MARS	BRT	MAXENT	RF
AUC	0.84 (0.81)	0.82 (0.81)	0.94 (0.93)	0.86 (0.84)	0.89 (0.86)
TSS	0.51 (0.50)	0.47 (0.46)	0.73 (0.70)	0.53 (0.50)	0.60 (0.59)
%Co	75.7 (73.9)	73.5 (73.2)	86.4 (84.4)	77.5 (74.2)	81.0 (80.1)

### 3.2 Current distribution models

**Figure 2:**
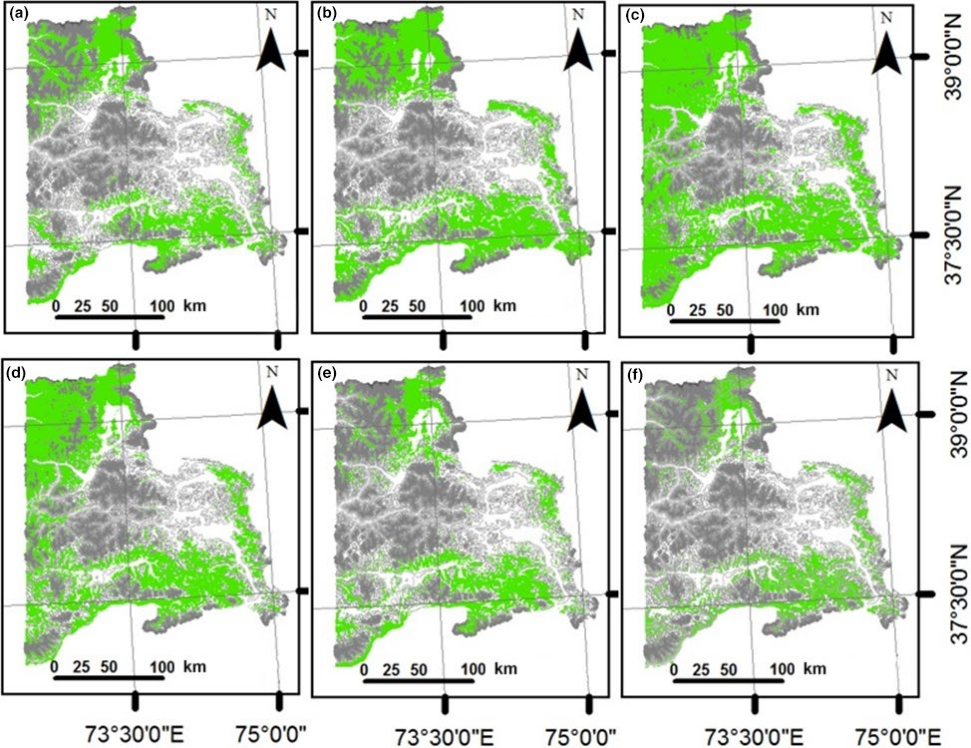
Current habitat suitability areas (in green) for the argali in eastern Tajikistan using climatic and nonclimatic variables in the models: (a) BRT, boosted regression tree, (b) GLM, generalized linear model, (c) MARS, multivariate adaptive regression splines, (d) Maxent, (e) RF, Random Forest, and (f) suitable area based on the agreement of at least three ENMs. Gray lines depict the contours in the region. The darker the lines, the higher the elevation

**Figure 3:**
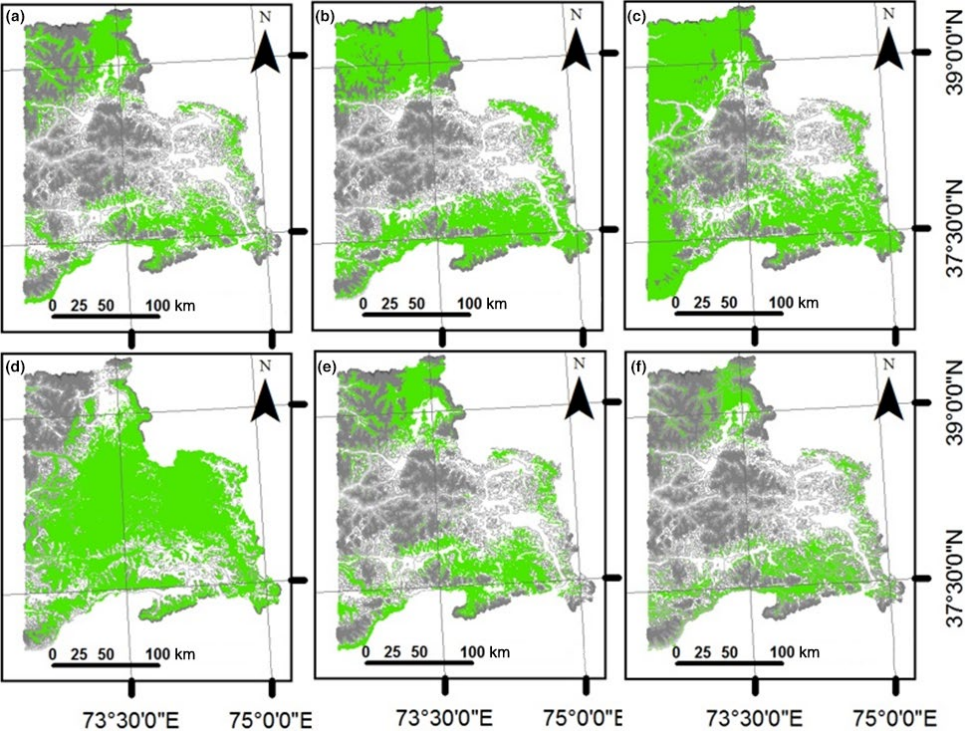
Current habitat suitability areas (in green) for the argali in eastern Tajikistan using only climatic variables in the models: (a) BRT, boosted regression tree, (b) GLM, generalized linear model, (c) MARS, multivariate adaptive regression splines, (d) Maxent, (e) RF, Random Forest, and (f) suitable area based on the agreement of at least three ENMs. Gray lines depict the contours in the region. The darker the lines, the higher the elevation

Using all variables, the five ENMs showed similar patterns of potential suitable conditions for argali in the northern and southeastern regions of the study area (Figure 2). Visual assessment of the maps revealed some differences, however. The five statistical models projected differing size of suitable areas even though they were using the same dataset of occurrence points. Among the five models, only RF and BRT (Figure 2a and e) identified potential suitable conditions in locations where presence points were mostly concentrated. These two suitability maps are associated with fairly high AUC values in Table 2 for RF (0.89) and BRT (0.94). GLM, MARS, and Maxent (Figure 2b, c, and d) highlighted suitable conditions in the western part of the study area, although MARS accentuated the suitable environmental conditions in the extreme eastern portion. The area of the current potential distribution of the argali, considering climatic and nonclimatic variables and based on the agreement of at least three ENMs, is 11,432 km^2^. Using climatic variables only, more locations were identified as potential suitable conditions for argali in all five models (Figure 3) compared to models that included the nonclimatic variables. The area of the current potential distribution of the argali, considering climatic variables only and based on the agreement of at least three ENMs, encompasses 13,242 km^2^. Similar to the results in Figure 2, only RF and BRT (Figure 3a and e) identified potential suitable conditions in locations where presence points were mostly concentrated.

### 3.3 Projecting models to future climatic conditions

**Figure 4:**
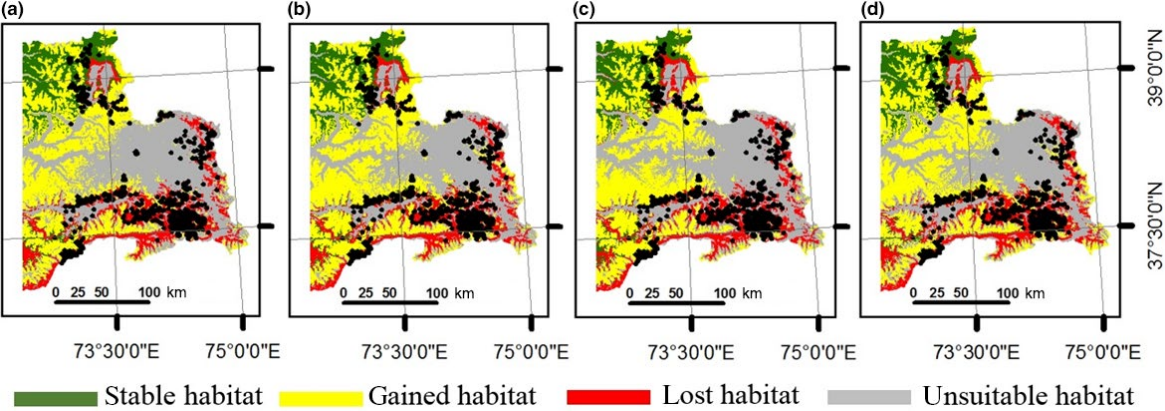
Comparison of suitable habitat distributions between present and future scenarios for Marco Polo argali in eastern Tajikistan using all environmental variables (climatic and nonclimatic). Future model was based on general circulation models projected according to: (a) RCP 4.5 to the year 2050; (b) RCP 8.5 to the year 2050; (c) RCP 4.5 to the year 2070; (d) RCP 8.5 to the year 2070. Maps show areas where present and future habitats agree (stable in green), future habitat projects new suitable conditions (gained in yellow), present suitable habitat may be converted to unsuitable in the future (lost in red), and areas where conditions are unsuitable now and in the future (unsuitable in gray). Black dots represent argali occurrence in our data sets. The entire map was generated using the tool of ArcGIS 10.2 (ESRI, Redlands, CA, USA, http://www.esri.com/)

**Figure 5:**
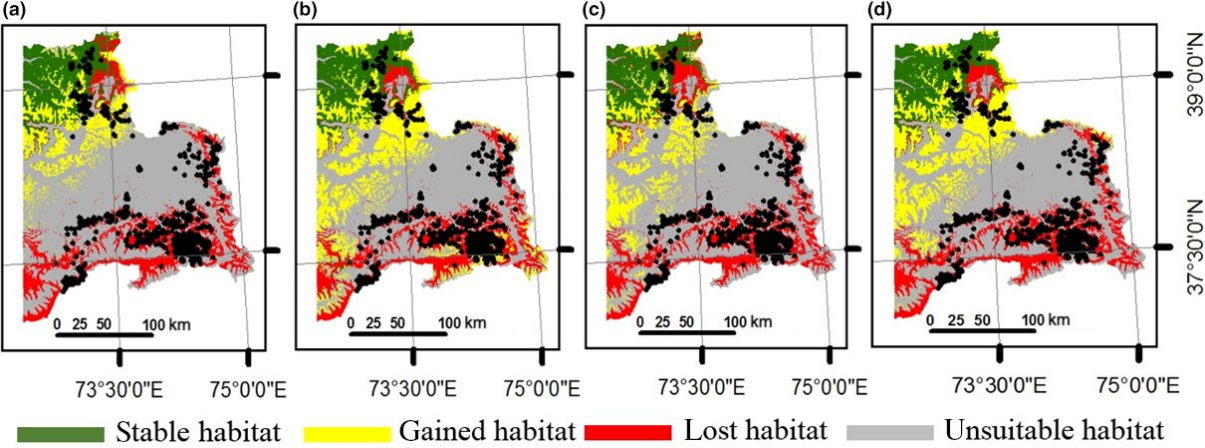
Comparison of suitable habitat distributions between present and future for the Marco Polo argali in eastern Tajikistan using only bioclimatic variables. Future model was based on general circulation models projected according to: (a) RCP 4.5 to the year 2050; (b) RCP 8.5 to the year 2050; (c) RCP 4.5 to the year 2070; (d) RCP 8.5 to the year 2070. Maps show areas where present and future habitats agree (stable in green), future habitat projects new suitable conditions (gained in yellow), present suitable habitat may be converted to unsuitable in the future (lost in red), and areas where conditions are unsuitable now and in the future (unsuitable in gray). Black dots represent argali occurrence in our data sets. The entire map was generated using the tool of ArcGIS 10.2 (ESRI, Redlands, CA, USA, http://www.esri.com/)

Effects of climate change for present suitable areas for argali are shown in Figures 4 and 5. These are maps of projected scenarios generated through comparison of ensembles of suitable conditions between present and projected future climatic conditions. These maps show areas where ensembles agree between present and future (stable), future ensemble projections of new suitable conditions (gain), and where present suitable ensemble projections may be converted to unsuitable conditions in the future (loss), and areas where conditions are unsuitable now and in the future (non). According to our projections for models using all environmental predictors, gains were observed even for the worst climate scenario—RCP 8.5 projected to year 2070 (Figure 4d). The average gains for 2050 and 2070 projections (Figure 4) were more than doubled the present suitable habitat (Table 3). Gains were also observed for models with climatic variables only (Figure 5). The total area containing suitable conditions (Figure 5a–d) increased from present to projected future, with an average gain of 46.3% by 2050 and 46.0% average gain by 2070 (Table 3).

**Table 3:** Areas (km^2^) and percentages of potential habitat stability, gain, and loss of Marco Polo argali for projections to 2050 and 2070 under climate change. Suitable habitat is considered stable when present and future ensemble maps agree that the area is suitable for the species. Gain in suitable habitat is recorded when projections find suitable habitat beyond the extent of the present suitable areas. There is loss of suitable habitat when present suitable areas become unsuitable in the future

Year	Climatic variables only			All variables		
	Average for RCPs 4.5 & 8.5			Average for RCPs 4.5 & 8.5		
	Stable (km^2^) (%)	Gain (km^2^) (%)	Lost (km^2^) (%)	Stable (km^2^) (%)	Gain (km^2^) (%)	Lost (km^2^) (%)
2050	4,895 (37.0)	6,126 (46.3)	8,347 (63.0)	4,504 (39.4)	13,485 (118.0)	6,928 (60.6)
2070	4,559 (34.4)	6,085 (46.0)	8,683 (65.6)	4,212 (36.9)	13,356 (116.8)	7,219 (63.2)

While gains were observed in new locations, the current areas of suitable habitat for the argali were projected to decline in 2050 and 2070 under all RCP scenarios (Figures 4 and 5). Majority of the losses were observed in the lower elevation (3,300 to 4,300 m) in the southeastern region of the study area where occurrence points were mostly concentrated. Models with climate variables predicted that 63.0% (8,347 km^2^) and 65.6% (8,683 km^2^) of present suitable argali habitat would be lost by 2050 and 2070, respectively (Table 3). Models that considered all environmental variables also predicted losses of 60.6% (6,928 km^2^) and 63.2% (7,219 km^2^) by 2050 and 2070, respectively. Fortunately, we observed current suitable areas located in higher elevations in the northern region (4,500–6,900 m) would continue to persist with future climate change. Also, we observed more habitat gains at much higher elevation in the study area, showing that elevational shift of habitat use could occur in the future. Total gains observed for models with all environmental variables were 118% (13,485 km^2^) for 2050 scenario and 116.8% (13,356 km^2^) for 2070 scenario.

In terms of habitat stability, the percentages of areas that would remain suitable for argali in 2050 and 2070 were a little higher for scenarios using climatic variables only than with scenarios using all environmental variables. For instance, 37.0% (4,895 km^2^) of current suitable areas would remain suitable in 2050 for models with climatic variables only, while those models that considered all predictors showed 39.4% (4,504 km^2^). The same could be deduced for projection results for 2070: 34.4% (4,559 km^2^) (climatic variables) and 36.9% (4,212 km^2^) (all variables).

This overestimation was likely the result of error of commission, where regions were considered climatically suitable in spite of underlying environmental conditions that make actual presence improbable (Sohl, 2014).

## 4 DISCUSSION

The effects of climate change are already being detected in the shifting and contraction of species’ ranges (Raxworthy, 2008; Sexton et al., 2009; Su, Aryal, Nan, & Ji, 2015), variations in species’ ecological interactions (IPCC 2007), and suboptimal movement behavior patterns (van Beest & Milner, 2013). This change could be deleterious (Lannoo, 2005), leading to reduction in species abundance as habitats deteriorate (Halpin, 1997). When global temperatures exceed 2.5°C, there could be negative consequences for biodiversity especially in montane habitats, where extensive species losses (up to 60% under high emission scenario by 2080) could occur (IPCC (Intergovernmental Panel on Climate Change), 2007). Further, the reduction in glacier thickness would have detrimental effects on many organisms, specifically on mammals and large predators (Haynes et al., 2014; IPCC (Intergovernmental Panel on Climate Change), 2007). In Tajikistan, the fauna of high mountain regions are the most sensitive to climate change (NCCA 2003), and could result in declines of rare and at‐risk species, such as argali. Our models indicated that climate change would have significant negative impacts on argali habitat in eastern Tajikistan.

### 4.1 Present and future projections

This is the first study to model climate change and map the current and future habitat suitability of argali in Tajikistan (and in Asia). This is also the first study to use an ensemble of modeling algorithms and climate models for a large mammal species endemic to the mountainous regions of Central Asia. We modeled the argali suitable habitats under current and future climates using two datasets, one with only climatic variables and another set that included other environmental variables. Models of present conditions were controlled by annual mean temperature, suggesting that this important climatic variable was a major limiting factor for argali. The association of argali to low temperatures was also reported in Khan et al. (2015), where it was labeled a major factor influencing species distribution and habitat suitability for ungulates in the Karakoram‐Pamir Landscape between China and Pakistan**.** Other studies also acknowledged the importance of temperature in modeling ungulates habitat suitability (Aryal, Raubenheimer, & Brunton, 2013; Forrest et al., 2012), although some used elevation as a substitute for temperature (Aryal, Brunton, Ji, et al., 2014). Apart from the annual mean temperature, we expected the precipitation data in the warmest quarter to rank high in the models, which our results confirmed. Other modeling studies for argali such Khan et al. (2015) did not include precipitation in the warmest quarter as a predictor. Abundant precipitation in dry months is key to moist meadows and forage development. St‐Louis and Côté (2014) found that forage quality can be a key factor determining habitat selection patterns for large herbivores. Likewise, the vegetation cover was found by our models to be an important contributor to the predicted habitat of Marco Polo argali. Salas et al. (2015) showed that the availability of forage and the distribution of argali are strongly correlated. Previous studies also reported cases where the normalized difference vegetation index (NDVI) (Guyot & Gu, 1994), used as an index of forage abundance (NDVI > 0.4) (Singh et al., 2009), was linked to the distribution of large herbivores (Pettorelli, 2014). The slope and elevation did not rank high on importance unlike previous studies showing them as major limiting factors for ungulates (Aryal, Brunton, Ji, et al., 2014; Forrest et al., 2012). However, terrain roughness was a significant predictor of habitat probably because the variable is, in part, defined by slope. Suitable areas for argali were found primarily on gentler slopes (0° to 15°), as also observed by Singh et al. (2009), Chetri and Pokharel (2005), and Namgali, Fox, and Bhatnagar (2004).

While there were differences in results among models, all future projections showed similar spatial patterns of habitat losses in 2050 and 2070, driven by climatic factors. The significant reduction in the present‐day distribution of suitable conditions for the argali would potentially occur at lower elevations in the southeastern Pamirs. The massive habitat loss could be attributed to the decrease in precipitation projected specifically for the lower elevation areas of southeastern Tajikistan (SHCOEP 2014). NCCA (2003) has documented the effects of climate change in the Tajikistan Pamirs with continuing glaciers degradation. In contrast, in the northern part of the study area where relatively high elevations occur, suitable habitats are projected to remain stable. On average, Marco Polo argali could lose more than two‐thirds of their present suitable habitat by 2070. Our results predicted higher estimates of loss than those ungulate studies conducted in other mountainous regions. For example, Luo et al. (2015) predicted more than half of the current suitable areas for the Tibetan ungulates would be lost by 2080 due to climate change. This difference in estimates could be because the Tibetan Plateau, compared to other mountain systems, is retreating at an accelerating rate due to global warming (IPCC (Intergovernmental Panel on Climate Change), 2007; Radi et al., 2014). Also, ungulates located in different regions of the mountain may have different habitat requirements and not equally sensitive to climate change (Chen, Hill, Ohlemuller, Roy, & Thomas, 2011).

Although our future models detected retention of suitable habitat in parts of the northern region, we still observed a shift of the species distribution northward (latitudinal) and possibly to higher elevations propelled by climate change, as the southern region experienced a major decline of suitable habitat. Previous research showed the spatial response to climate change for a variety of species—shifting distributions northward and upward (Alvarez, Salas, Harings, & Boykin, 2017; Ogawa‐Onishi, Berry, & Tanaka, 2010; Popy, Bordignon, & Prodon, 2010; Root et al., 2003; Wilson et al., 2005). In our results, about 20% (2,500 km^2^) of the average habitat gains in 2070 showed a shift northwards. In contrast to Luo et al. (2015) that detected no elevational shift of Tibetan ungulates, our model projected a future altitudinal change of suitable areas from lower to higher elevations in the northern region of the study area where the highest elevations occur. An elevational shift of forage productivity to higher latitudes (Singh, Grachev, Bekenov, & Milner‐Gulland, 2010) could result in altitudinal shifting of argali in response to climate change.

### 4.2 Uncertainty and robustness of future projections

Three have been objections by a handful of studies about the use of climatic variables alone to model the climate envelope of species (Geyer, 2011; Sax, 2007) because there are a wide range of climate change‐related stresses that are at play that could affect population ecology and physiology. Climatic variables alone may not always correctly ascertain current or future species distribution over space (Salas, Seamster, et al., 2017). Despite the limitations of models using climatic variables solely, several proponents have praised their crucial role to provide broad insights on the future effects of climate change on species distribution (Guisan & Thuiller, 2005; Soberón, 2007). Although complete assessment of the effects of climate change on argali includes other indirect variables such herders’ presence and general human presence (Panthi, Khanal, Acharya, Aryal, & Srivathsa, 2017; Shrestha et al., 2014) (including poaching pressure) or predicted paved roads and villages, they are not often utilized when projecting to future distributions. Also, a study by Bucklin et al. (2015) using 14 vertebrate species found that climate variables were more critical in ENMs than other environmental predictors. Our models used nonclimatic dataset that were found to optimize model performance and ecological applicability. However, variables that would have added value to the current study, such as species life history, dispersal capacity, and the complex ecological interrelationships among species (Soberón, 2007), were not integrated into the modeling process due to data unavailability. Furthermore, when we projected present conditions to future years, we assumed that the land cover and other topographic factors in the region would remain stable in the future. These shortcomings of our models could result to spatial mismatch between the projected future and real distributions of argali habitats. Results of our study may not deliver the most accurate predicted distributions; however, they could still facilitate in Marco Polo argali conservation planning in the region and provide approximation of changes of species distribution in the context of climate change.

In terms of our modeling techniques, multiple limitations associated with the projection of species distributions into the future under different climate scenarios have been documented. Three broad categories of uncertainties affecting the climate variables used to drive the ENMs include (a) uncertainties in future greenhouse gas concentrations (Meinshausen, 2011), (b) limitations in the accuracy of GCM‐simulated, large‐scale physical climate responses to changing greenhouse gas levels (Knutti & Sedláček, 2013), and (c) shortcomings and assumptions inherent to statistical downscaling methods used to refine GCM results to a finer level of spatial detail (Barsugli, 2013; Dixon, 2016). By utilizing data products derived from four GCMs and two RCPs, this study partially explores two of these three sources of climate variable uncertainty. Stoklosa, Daly, Foster, Ashcroft, and Warton (2015) specifically discuss approaches to account for some uncertainties in the climate variables used to drive ENMs. Furthermore, several authors have shown variability among future projections of suitable climatic conditions when using different climate models applied to the same species occurrence dataset (Bakkenes, Alkemade, & Ihle, 2002).

Variability of results makes assessment of projections of future conditions a complex effort. First, it is not possible to determine which single ENM could provide the most accurate information for a species, although one could argue that the climate model with the highest accuracy in capturing the present‐day suitable habitat conditions may produce more accurate future projections. However, Thuiller (2004) reasoned that even when a model has the highest AUC and K statistics, the model may not provide the best estimate of the future distribution of suitable conditions, especially as every model is based on different assumptions. It is most fitting to use an ensemble model of future projections, as this ensemble represents the areas of agreement among individual model projections. The reliability of future conditions produced by ensembles may be questioned, but ensemble results do incorporate the positive aspects of multiple models and provide a more conservative assessment of these conditions.

## 5 CONCLUSION

The main contribution of this study is the quantification of the possible impact of climate change regimes on the availability of future suitable habitats for Marco Polo argali. Results in all our models showed major losses of suitable habitat for the populations of argali in eastern Tajikistan. While our results are potential projections of future Marco Polo argali suitable habitat, this is the first quantitative assessment of habitat shifts of argali under future climate change in eastern Tajikistan, which could provide information for conservation planning for this near threatened species in the region. There is a need to conduct a similar study to include habitats in adjacent populations in Afghanistan, China, Pakistan, and Kyrgyzstan and to determine the extent of changes in critical habitats in climate scenarios.

## CONFLICT OF INTEREST

None declared.

## AUTHOR CONTRIBUTION

Eric Ariel Salas conceived and designed the research; performed the modeling; analyzed and interpreted the data; contributed reagents, materials, analysis tools or data; and wrote the manuscript. Raul Valdez interpreted the data and contributed reagents, materials, analysis tools, or data. Stefan Michel contributed reagents, materials, analysis tools, or data. Kenneth Boykin contributed reagents, materials, analysis tools, or data.

## References

[ece34103-bib-0001] Allouche, O. , Tsoar, A. , & Kadmon, R. (2006). Assessing the accuracy of species distribution models: Prevalence, Kappa and the True Skill Statistic (TSS). Journal of Applied Ecology, 43, 1223–1232. https://doi.org/10.1111/j.1365-2664.2006.01214.x

[ece34103-bib-0002] Alvarez, G. , Salas, E. A. L. , Harings, N. M. , & Boykin, K. G. (2017). Projections of future suitable bioclimatic conditions of parthenogenetic whiptails. Climate, 5, 34 https://doi.org/10.3390/cli5020034

[ece34103-bib-0003] Araújo, M. B. , Whittaker, R. J. , Ladle, R. J. , & Erhard, M. (2005). Reducing uncertainty in projections of extinction risk from climate change. Global Ecology and Biogeography, 5, 529–538. https://doi.org/10.1111/j.1466-822X.2005.00182.x

[ece34103-bib-0004] Arora, V. K. , Scinocca, J. F. , Boer, G. J. , Christian, J. R. , Denman, K. L. , Flato, G. M. , … Merryfiel, W. G. (2011). Carbon emission limits required to satisfy future representative concentration pathways of greenhouse gases. Geophysical Research Letters, 38, L05805.

[ece34103-bib-0005] Aryal, A. , Brunton, D. , Ji, W. , Karmacharya, D. , McCarthy, T. , & Bencini, R. (2014). Multipronged strategy including genetic analysis for assessing conservation options for the snow leopard in the central Himalaya. Journal of Mammalogy, 95, 871–881. https://doi.org/10.1644/13-MAMM-A-243

[ece34103-bib-0006] Aryal, A. , Brunton, D. , & Raubenheimer, D. (2014). Impacts of climate change on human‐wildlife‐ecosystem interactions in the Trans‐Himalayan region of Nepal. Theoretical and Applied Climatology, 115, 517–529. https://doi.org/10.1007/s00704-013-0902-4

[ece34103-bib-0007] Aryal, A. , Raubenheimer, D. , & Brunton, D. (2013). Habitat assessment for the translocation of blue sheep to maintain a viable snow leopard population in the Mt Everest Region, Nepal. Zoology and Ecology, 23, 66–82. https://doi.org/10.1080/21658005.2013.765634

[ece34103-bib-0008] Badenkov, Y.P. (1992). Mountains of the former Soviet Union: Value, diversity, uncertainty In Stone P.D. (Ed.), The state of the world's mountains. A global report (pp. 257–299). London and New Jersey: Zed Books.

[ece34103-bib-0009] Bakkenes, M. , Alkemade, J. R. M. , & Ihle, F. (2002). Assessing effects of forecasted climate change on the diversity and distribution of European higher plants for 2050. Global Change Biology, 8, 390–407. https://doi.org/10.1046/j.1354-1013.2001.00467.x

[ece34103-bib-0010] Barsugli, J. J. (2013). The practitioner's dilemma: How to assess the credibility of downscaled climate projections. Eos, Transactions, American Geophysical Union, 94, 424–425. https://doi.org/10.1002/2013EO460005

[ece34103-bib-0011] van Beest, F. M. , & Milner, J. M. (2013). Behavioural responses to thermal conditions affect seasonal mass change in a heat‐sensitive northern ungulate. PLoS ONE, 8(6), e65972 https://doi.org/10.1371/journal.pone.0065972 23776584 10.1371/journal.pone.0065972 PMC3679019

[ece34103-bib-0012] BIOFOR Chemonics International Inc. (2001). Biodiversity assessment for Tajikistan task order under the Biodiversity & Sustainable Forestry IQC (BIOFOR). Retrieved from http://www.biofor.com/documents/Tajikistan.pdf

[ece34103-bib-0013] Breiman, L. (2001). Random forests. Machine Learning, 45, 5–32. https://doi.org/10.1023/A:1010933404324

[ece34103-bib-0014] Breu, T. , & Hurni, H. (2003). The Tajik Pamirs: Challenges of sustainable development in an isolated mountain region (p. 80). Switzerland: Centre for Development and Environment (CDE), University of Berne, Berne.

[ece34103-bib-0015] Bucklin, D. N. , Basille, M. , Benscoter, A. M. , Brandt, L. A. , Mazzotti, F. J. , Romañach, S. S. , … Watling, J. I. (2015). Comparing species distribution models constructed with different subsets of environmental predictors. Diversity and Distributions, 21, 23–35. https://doi.org/10.1111/ddi.12247

[ece34103-bib-0016] Chen, I. C. , Hill, J. K. , Ohlemuller, R. , Roy, D. B. , & Thomas, C. D. (2011). Rapid range shifts of species associated with high levels of climate warming. Science, 333, 1024–1026. https://doi.org/10.1126/science.1206432 21852500 10.1126/science.1206432

[ece34103-bib-0017] Chetri, M. , & Pokharel, A. (2005). Status and distribution of blue sheep, Tibetan argali and the kiang in Damodar Kunda area, Upper Mustang, Nepal. Our Nature, 3, 56–62.

[ece34103-bib-0018] Cohen, J. (1960). A coefficient of agreement for nominal scales. Educational and Psychological Measurement, 20, 37–46. https://doi.org/10.1177/001316446002000104

[ece34103-bib-0019] Collins, W. J. , Bellouin, N. , Doutriaux‐Boucher, M. , Gedney, N. , Halloran, P. , Hinton, T. , … Liddicoat, S. (2011). Development and evaluation of an Earth‐System model – HadGEM2. Geoscientific Model Development, 4, 1051–1075. https://doi.org/10.5194/gmd-4-1051-2011

[ece34103-bib-0020] Dixon, K. W. (2016). Evaluating the stationarity assumption in statistically downscaled climate projections: is past performance an indicator of future results? Climatic Change, 135, 395–408. https://doi.org/10.1007/s10584-016-1598-0

[ece34103-bib-0021] Donner, L. J. , Wyman, B. L. , Hemler, R. S. , Horowitz, L. W. , Ming, Y. , Zhao, M. , … Zeng, F. (2011). The dynamical core, physical parameterizations, and basic simulation characteristics of the atmospheric component AM3 of the GFDL Global Coupled Model CM3. Journal of Climate, 24, 3484–3519. https://doi.org/10.1175/2011JCLI3955.1

[ece34103-bib-0022] Dormann, C. F. , Jane, E. , Bacher, S. , Buchmann, C. , Carl, G. , Carré, G. , … Leitao, P. J. (2013). Collinearity: a review of methods to deal with it and a simulation study evaluating their performance. Ecography, 36, 27–46. https://doi.org/10.1111/j.1600-0587.2012.07348.x

[ece34103-bib-0023] Elith, J. , Catherine, H. G. , Dudík, M. , Ferrier, S. , Guisan, A. , Hijmans, R. J. , & Huettmann, F. (2006). Novel methods improve prediction of species’ distributions from occurrence data. Ecography, 29, 129–151. https://doi.org/10.1111/j.2006.0906-7590.04596.x

[ece34103-bib-0024] Elith, J. , & Leathwick, J. R. (2009). Species distribution models: ecological explanation and prediction across space and time. Annual Review of Ecology, Evolution, and Systematics, 40, 677–697. https://doi.org/10.1146/annurev.ecolsys.110308.120159

[ece34103-bib-0025] Elith, J. , Leathwick, J. R. , & Hastie, T. (2008). A working guide to boosted regression trees. Journal of Animal Ecology, 77, 802–813. https://doi.org/10.1111/j.1365-2656.2008.01390.x 18397250 10.1111/j.1365-2656.2008.01390.x

[ece34103-bib-0026] Fielding, A. H. , & Bell, J. F. (1997). A review of methods for the assessment of prediction errors in conservation presence/absence models. Environmental Conservation, 24, 38–49. https://doi.org/10.1017/S0376892997000088

[ece34103-bib-0027] Finaev, A. F. , Liu, S. , Bao, W. J. , & Li, J. (2016). Climate Change and water potential of the Pamir Mountains. Geography, Environment, Sustainability, 9, 88–105. https://doi.org/10.15356/2071-9388

[ece34103-bib-0028] Forrest, J. L. , Wikramanayake, E. , Shrestha, R. , Areendran, G. , Gyeltshen, K. , Maheshwari, A. , … Thapa, K. (2012). Conservation and climate change: assessing the vulnerability of snow leopard habitat to treeline shift in the Himalaya. Biological Conservation, 150, 129–135. https://doi.org/10.1016/j.biocon.2012.03.001

[ece34103-bib-0029] Fuller, A. , Mitchell, D. , Maloney, S. K. , & Hetem, R. (2016). Towards a mechanistic understanding of the responses of large terrestrial mammals to heat and aridity associated with climate change. Climate Change Responses, 3, 10 https://doi.org/10.1186/s40665-016-0024-1

[ece34103-bib-0030] Gama, M. , Crespo, D. , Dolbeth, M. , & Anastácio, P. M. (2015). Predicting global habitat suitability for Corbicula fluminea using species distribution models: The importance of different environmental datasets. Ecological Modelling, 319, 163–169.

[ece34103-bib-0031] Gent, P. R. , Danabasoglu, G. , Donner, L. J. , Holland, M. M. , Hunke, E. C. , Jayne, S. R. , … Zhang, M. (2011). The community climate system model version 4. Journal of Climate, 24, 4973–4991. https://doi.org/10.1175/2011JCLI4083.1

[ece34103-bib-0032] Geyer, J. (2011). Classification of climate‐change‐induced stresses on biological diversity. Conservation Biology, 25, 708–715. https://doi.org/10.1111/j.1523-1739.2011.01676.x 21488958 10.1111/j.1523-1739.2011.01676.x

[ece34103-bib-0033] Granados, A. , & Brodie, J. F. (2016). Persistence of tropical Asian ungulates in the face of hunting and climate change In Sankaran M., & Ahrestani F. (Eds.), The ecology of large herbivores in South and Southeast Asia (pp. 223–235). Berlin, Germany: Springer‐Verlag https://doi.org/10.1007/978-94-017-7570-0

[ece34103-bib-0034] Guisan, A. , & Thuiller, W. (2005). Predicting species distribution: offering more than simple habitat models. Ecological Letters, 8, 993–1009. https://doi.org/10.1111/j.1461-0248.2005.00792.x 10.1111/j.1461-0248.2005.00792.x 34517687

[ece34103-bib-0035] Guyot, G. , & Gu, X. F. (1994). Effect of radiometric corrections on NDVI determined from SPOT‐HRV and Landsat‐TM data. Remote Sensing of Environment, 49, 169–180. https://doi.org/10.1016/0034-4257(94)90012-4

[ece34103-bib-0036] Halpin, P. N. (1997). Global climate change and natural‐area protection: management responses and research directions. Ecological Applications, 7, 828–843. https://doi.org/10.1890/1051-0761(1997)007[0828:GCCANA]2.0.CO;2

[ece34103-bib-0037] Hansen, P. J. (2009). Effects of heat stress on mammalian reproduction. Philosophical Transactions of the Royal Society of London, 364, 3341–3350. https://doi.org/10.1098/rstb.2009.0131 19833646 10.1098/rstb.2009.0131 PMC2781849

[ece34103-bib-0038] Harris, R.B. , & Reading, R. (2008). Ovis ammon. In IUCN 2008. The IUCN Red List of Threatened Species. Version 2012.1. http://www.iucnredlist.org. Accessed 18 June 2017.

[ece34103-bib-0039] Haslinger, A. , Breu, T. , Hurni, H. , & Maselli, D. (2007). Opportunities and risks in reconciling conservation and development in a post‐Soviet setting: The example of the Tajik National Park. International Journal of Biodiversity Science and Management, 3, 157–169. https://doi.org/10.1080/17451590709618170

[ece34103-bib-0040] Haynes, M. A. , Kung, K. S. , Brandt, J. S. , Yongping, Y. , & Waller, D. M. (2014). Accelerated climate change and its potential impact on yak herding livelihoods in the eastern Tibetan plateau. Climatic Change, 123, 147–160. https://doi.org/10.1007/s10584-013-1043-6

[ece34103-bib-0041] Hijmans, R. J. , Cameron, S. E. , Parra, J. L. , Jones, P. G. , & Jarvis, A. (2005). Very high resolution interpolated climate surfaces for global land areas. International Journal of Climatology, 25, 1965–1978. https://doi.org/10.1002/(ISSN)1097-0088

[ece34103-bib-0042] Hole, D. G. , Willis, S. G. , Pain, D. J. , Fishpool, L. D. , Butchart, S. H. M. , Collingham, Y. C. , … Huntley, B. (2009). Projected impacts of climate change on a continent‐wide protected area network. Ecology Letters, 12, 420–431. https://doi.org/10.1111/j.1461-0248.2009.01297.x 19379136 10.1111/j.1461-0248.2009.01297.x

[ece34103-bib-0043] IPCC (Intergovernmental Panel on Climate Change) (2007). Climate Change 2007: IPCC Fourth Assessment Report (p. 112). Cambridge, UK: Cambridge University Press.

[ece34103-bib-0044] Jacobson, A. R. , Provenzale, A. , von Hardenberg, A. , Bassano, B. , & Festa‐Bianchet, M. (2004). Climate forcing and density dependence in a mountain ungulate population. Ecology, 85, 1598–1610. https://doi.org/10.1890/02-0753

[ece34103-bib-0045] Jessen, C. (2001). Temperature regulation in humans and other mammals. Journal of Animal Physiology and Animal Nutrition, 85, 333 https://doi.org/10.1046/j.1439-0396.2001.0335a.x

[ece34103-bib-0046] Khan, B. , Ablimit, A. , Khan, G. , Jasra, A. W. , Ali, H. , Ali, R. , … Ismail, M. (2015). Abundance, distribution and conservation status of Siberian ibex, Marco Polo and blue sheep in Karakoram‐Pamir Mountain area. Journal of King Saud University –Science, 28, 216–225.

[ece34103-bib-0047] Khromova, T. E. , Osipova, G. B. , Tsvetkov, D. G. , Dyurgerov, M. B. , & Barry, R. G. (2006). Changes in glacier extent in the eastern Pamir, Central Asia, determined from historical data and Aster imagery. Remote Sensing of Environment, 102, 24–32. https://doi.org/10.1016/j.rse.2006.01.019

[ece34103-bib-0048] Knutti, R. , & Sedláček, J. (2013). Robustness and uncertainties in the new CMIP5 climate model projections. Nature Climate Change, 3, 369–373. https://doi.org/10.1038/nclimate1716

[ece34103-bib-0049] Lamsal, P. , Kumar, L. , Aryal, A. , & Atreya, K. (2018). Future climate and habitat distribution of Himalayan Musk Deer (*Moschus chrysogaster*). Ecological Informatics, 44, 101–108. https://doi.org/10.1016/j.ecoinf.2018.02.004

[ece34103-bib-0050] Lannoo, M. J. (2005). Amphibian declines: The conservation status of United States species (p. 1115). Berkeley, CA: University of California Press https://doi.org/10.1525/california/9780520235922.001.0001

[ece34103-bib-0051] Leathwick, J. R. , Elith, J. , & Hastie, T. (2006). Comparative performance of generalized additive models and multivariate adaptive regression splines for statistical modelling of species distributions. Ecological Modelling, 199, 188–196. https://doi.org/10.1016/j.ecolmodel.2006.05.022

[ece34103-bib-0052] Liaw, A. , & Wiener, M. (2002). Classification and regression by randomForest. R News, 2, 182.

[ece34103-bib-0053] Liu, C. , Berry, P. M. , Dawson, T. P. , & Pearson, R. G. (2005). Selecting thresholds of occurrence in the prediction of species distributions. Ecography, 28, 385–393. https://doi.org/10.1111/j.0906-7590.2005.03957.x

[ece34103-bib-0054] Lobo, J. M. , Jiménez‐Valverde, A. , & Real, R. (2008). AUC: A Misleading measure of the performance of predictive distribution models. Global Ecology and Biogeography, 17, 145–151. https://doi.org/10.1111/j.1466-8238.2007.00358.x

[ece34103-bib-0055] Luo, Z. , Jiang, Z. , & Tang, S. (2015). Impacts of climate change on distributions and diversity of ungulates on the Tibetan Plateau. Ecological Applications, 25(1), 24–38. https://doi.org/10.1890/13-1499.1 26255355 10.1890/13-1499.1

[ece34103-bib-0056] Manel, S. , Williams, H. C. , & Ormerod, S. J. (2001). Evaluating presence‐absence models in ecology: the need to account for prevalence. Journal of Applied Ecology, 38, 921–931.

[ece34103-bib-0057] Marmion, M. , Parviainen, M. , Luoto, M. , Heikkinen, R. K. , & Thuiller, W. (2009). Evaluation of consensus methods in predictive species distribution modelling. Diversity and Distributions, 15, 59–69. https://doi.org/10.1111/j.1472-4642.2008.00491.x

[ece34103-bib-0058] Meinshausen, M. (2011). The RCP greenhouse gas concentrations and their extensions from 1765 to 2300. Climatic Change, 109, 213 https://doi.org/10.1007/s10584-011-0156-z

[ece34103-bib-0059] Meller, L. , Cabeza, M. , Pironon, S. , Barbet‐Massin, M. , Maiorano, L. , Georges, D. , & Thuiller, W. (2014). Ensemble distribution models in conservation prioritization: from consensus predictions to consensus reserve networks. Diversity & Distributions, 20, 309–321. https://doi.org/10.1111/ddi.12162 24791145 10.1111/ddi.12162 PMC4003394

[ece34103-bib-0060] Merzlyakova, I. (2002). The mountains of central Asia and Kazakhstan In Shahgedanova M. (Ed.), The physical geography of Northern Eurasia (pp. 377–402). New York, NY: Oxford University Press.

[ece34103-bib-0061] Michel, S. , & Muratov, R. (2010). Survey of Marco Polo sheep and other mammal species in the eastern Pamirs Republic of Tajikistan, GBAO. Dushanbe, Tajikistan: Committee for Environmental Protection and GTZ.

[ece34103-bib-0062] Morisette, J. T. , Jarnevich, C. S. , Holcombe, T. R. , Talbert, C. B. , Ignizio, D. , Talbert, M. K. , … Young, N. E. (2013). VisTrails SAHM: Visualization and workflow management for species habitat modeling. Ecography, 36, 129–135. https://doi.org/10.1111/j.1600-0587.2012.07815.x

[ece34103-bib-0063] Namgali, T. , Fox, J. L. , & Bhatnagar, Y. V. (2004). Habitat segregation between sympatric Tibetan argali *Ovis ammon hodgsoni* and blue sheep *Pseudois nayaur* in the Indian Trans‐Himalaya. Journal of Zoology, 262, 57–63. https://doi.org/10.1017/S0952836903004394

[ece34103-bib-0064] NCCA (2003). National action plan of the Republic Tajikistan for climate change mitigation, Tajik met service (p. 234). Dushanbe, Tajikistan: NCCA.

[ece34103-bib-0065] Nogués‐Bravo, D. , Araujo, M. B. , Errea, M. P. , & Martinez‐Rica, J. P. (2007). Exposure of global mountain systems to climate warming during the 21st Century. Global Environmental Change, 17, 420–428. https://doi.org/10.1016/j.gloenvcha.2006.11.007

[ece34103-bib-0066] Ogawa‐Onishi, Y. , Berry, P. M. , & Tanaka, N. (2010). Assessing the potential impacts of climate change and their conservation implications in Japan: a case study of conifers. Biological Conservation, 143, 1728–1736. https://doi.org/10.1016/j.biocon.2010.04.021

[ece34103-bib-0067] Panthi, S. , Khanal, G. , Acharya, K. P. , Aryal, A. , & Srivathsa, A. (2017). Large anthropogenic impacts on a charismatic small carnivore: Insights from distribution surveys of red panda *Ailurus fulgens* in Nepal. PLoS ONE, 12, e0180978 https://doi.org/10.1371/journal.pone.0180978 28708881 10.1371/journal.pone.0180978 PMC5510994

[ece34103-bib-0068] Peterson, A. T. , Soberón, J. , Pearson, R. G. , Anderson, R. P. , Martínez‐Meyer, E. , Nakamura, M. , & Araujo, M. B. (2011). Ecological niches and geographic distributions (p. 328). Princeton, New Jersey: Princeton University Press.

[ece34103-bib-0069] Pettorelli, N. (2014). The normalized difference vegetation index (p. 194). New York, NY: Oxford University Press.

[ece34103-bib-0070] Pettorelli, N. , Pelletier, F. , von Hardenberg, A. , Festa‐Bianchet, M. , & Coté, S. D. (2007). Early onset of vegetation growth vs. rapid green‐up: Impacts on juvenile mountain ungulates. Ecology, 88, 381–390. https://doi.org/10.1890/06-0875 17479756 10.1890/06-0875

[ece34103-bib-0071] Phillips, S. J. , Anderson, R. P. , & Schapire, R. E. (2006). Maximum entropy modeling of species geographic distributions. Ecological Modelling, 190, 231–259. https://doi.org/10.1016/j.ecolmodel.2005.03.026

[ece34103-bib-0072] Phillips, S. J. , & Dudík, M. (2008). Modeling of species distributions with Maxent: new extensions and a comprehensive evaluation. Ecography, 31, 161–175. https://doi.org/10.1111/j.0906-7590.2008.5203.x

[ece34103-bib-0073] Popy, S. , Bordignon, L. , & Prodon, R. (2010). A weak upward elevational shift in the distributions of breeding birds in the Italian Alps. Journal of Biogeography, 37, 57–67.

[ece34103-bib-0074] Radi, V. , Bliss, A. , Beedlow, A. C. , Hock, R. , Miles, E. , & Cogley, G. (2014). Regional and global projections of twenty‐first century glacier mass changes in response to climate scenarios from global climate models. Climate Dynamics, 42, 37–58. https://doi.org/10.1007/s00382-013-1719-7

[ece34103-bib-0075] Raxworthy, C. J. (2008). Extinction vulnerability of tropical montane endemism from warming and upslope displacement: a preliminary appraisal for the highest massif in Madagascar. Global Change Biology, 14, 1703–1720. https://doi.org/10.1111/j.1365-2486.2008.01596.x

[ece34103-bib-0076] Rehfeldt, G. E. , Crookston, N. L. , Sáenz‐Romero, C. , & Campbell, E. M. (2012). North American vegetation model for land‐use planning in a changing climate: A solution to large classification problems. Ecological Applications, 22, 119–141. https://doi.org/10.1890/11-0495.1 22471079 10.1890/11-0495.1

[ece34103-bib-0077] Roeckner, E. , Giorgetta, M. A. , Crueger, T. , Esch, M. , & Pongratz, J. (2011). Historical and future anthropogenic emission pathways derived from coupled climate–carbon cycle simulations. Climatic Change, 105, 91–108. https://doi.org/10.1007/s10584-010-9886-6

[ece34103-bib-0078] Root, T. L. , Price, J. T. , Hall, K. R. , Schneider, S. H. , Rosenzweig, C. , & Pounds, J. A. (2003). Fingerprints of global warming on wild animals and plants. Nature, 421, 57–60. https://doi.org/10.1038/nature01333 12511952 10.1038/nature01333

[ece34103-bib-0079] Salas, E. A. L. , Boykin, K. G. , & Valdez, R. (2016). Multispectral and texture feature application in image‐object analysis of summer vegetation in eastern Tajikistan Pamirs. Remote Sensing, 8, 78 https://doi.org/10.3390/rs8010078

[ece34103-bib-0080] Salas, E. A. L. , Seamster, V. A. , Boykin, K. G. , Harings, N. M. , & Dixon, K. W. (2017). Modeling the impacts of climate change on Species of Concern (birds) in South Central USA based on bioclimatic variables. AIMS Environmental Science, 4, 358–385. https://doi.org/10.3934/environsci.2017.2.358

[ece34103-bib-0081] Salas, E. A. L. , Valdez, R. , & Boykin, K. G. (2015). Geographic layers as landscape drivers for the Marco Polo argali habitat in the southeastern Pamir Mountains of Tajikistan. ISPRS International Journal of Geo‐information, 4, 2094–2108. https://doi.org/10.3390/ijgi4042094

[ece34103-bib-0082] Salas, E. A. L. , Valdez, R. , & Michel, S. (2017). Summer and winter habitat suitability of Marco Polo argali in southeastern Tajikistan: A modeling approach. Heliyon, 3(11), e00445 https://doi.org/10.1016/j.heliyon.2017.e00445 29159323 10.1016/j.heliyon.2017.e00445 PMC5681343

[ece34103-bib-0083] Sappington, J. M. , Longshore, K. M. , & Thomson, D. B. (2007). Quantifying landscape ruggedness for animal habitat analysis: a case study using bighorn sheep in the Mojave Desert. Journal of Wildlife Management, 71, 1419–1426. https://doi.org/10.2193/2005-723

[ece34103-bib-0084] Sax, D. F. (2007). Ecological and evolutionary insights from species invasions. Trends in Ecology & Evolution, 22, 465–471. https://doi.org/10.1016/j.tree.2007.06.009 17640765 10.1016/j.tree.2007.06.009

[ece34103-bib-0085] Schaller, G. B. (1998). Wildlife of the Tibetan Steppe (p. 392). Chicago, IL: The University of Chicago Press.

[ece34103-bib-0086] Sexton, J. P. , McIntyre, P. J. , Angert, A. L. , & Rice, K. J. (2009). Evolution and ecology of species range limits. Annual Review of Ecology, Evolution, and Systematics, 40, 415–436. https://doi.org/10.1146/annurev.ecolsys.110308.120317

[ece34103-bib-0087] SHCOEP (State Administration for Hydrometeorology Committee on Environmental Protection) (2014). Tajikistan third national communication of the Republic of Tajikistan under the United Nations framework convention on climate change. Dushanbe, Tajikistan: SHCOEP (State Administration for Hydrometeorology Committee on Environmental Protection), p. 155.

[ece34103-bib-0088] Shen, G. Z. , Pimm, S. L. , Feng, C. , Ren, G. , Liu, Y. , Xu, W. , … Xie, Z. (2015). Climate change challenges the current conservation strategy for the giant panda. Biological Conservation, 190, 43–50. https://doi.org/10.1016/j.biocon.2015.05.004

[ece34103-bib-0089] Shrestha, T. , Aryal, A. , Rai, R. , Koirala, S. , Jnawali, D. , Kafle, R. , … Raubenheimer, D. (2014). Balancing wildlife and human needs: Protected forest approach in Nepal. Natural Areas Journal, 34, 376–380. https://doi.org/10.3375/043.034.0313

[ece34103-bib-0090] Singh, N. J. , Grachev, I. A. , Bekenov, A. B. , & Milner‐Gulland, E. J. (2010). Tracking greenery across a latitudinal gradient in central Asia –the migration of the saiga antelope. Diversity and Distributions, 16, 663–675. https://doi.org/10.1111/j.1472-4642.2010.00671.x

[ece34103-bib-0091] Singh, N. J. , Yoccoz, N. G. , Bhatnagar, Y. V. , & Fox, J. L. (2009). Using habitat suitability models to sample rare species in high‐altitude ecosystems: a case study with Tibetan argali. Biological Conservation, 18, 2893–2908.

[ece34103-bib-0092] Smith, T. S. , Flinders, J. T. , & Winn, D. S. (1991). A habitat evaluation procedure for Rocky Mountain bighorn sheep in the intermountain west. Great Basin National, 51, 205–225.

[ece34103-bib-0093] Soberón, J. (2007). Grinnellian and Eltonian niches and geographic distributions of species. Ecological Letters, 10, 1115–1123. https://doi.org/10.1111/j.1461-0248.2007.01107.x 10.1111/j.1461-0248.2007.01107.x 17850335

[ece34103-bib-0094] Sohl, T. L. (2014). The relative impacts of climate and land‐use change on conterminous United States bird species from 2001 to 2075. PLoS ONE, 9(11), e112251 https://doi.org/10.1371/journal.pone.0112251 25372571 10.1371/journal.pone.0112251 PMC4221285

[ece34103-bib-0095] St‐Louis, A. , & Côté, S. D. (2014). Resource selection in a high‐altitude rangeland equid, the kiang (Equus kiang): influence of forage abundance and quality at multiple spatial scales. Canadian Journal of Zoology, 92, 239–249. https://doi.org/10.1139/cjz-2013-0191

[ece34103-bib-0096] Stohlgren, T. J. , Ma, P. , Kumar, S. , Rocca, M. , Morisette, J. T. , Jarnevich, C. S. , & Benson, N. (2010). Ensemble habitat mapping of invasive plant species. Risk Analysis, 30, 224–235. https://doi.org/10.1111/j.1539-6924.2009.01343.x 20136746 10.1111/j.1539-6924.2009.01343.x

[ece34103-bib-0097] Stoklosa, J. , Daly, C. , Foster, S. D. , Ashcroft, M. B. , & Warton, D. I. (2015). A climate of uncertainty: accounting for error in climate variables for species distribution models. Methods in Ecology and Evolution, 6, 412–423. https://doi.org/10.1111/2041-210X.12217

[ece34103-bib-0098] Su, J. , Aryal, A. , Nan, Z. , & Ji, W. (2015). Climate change‐induced range expansion of a subterranean rodent: Implications for rangeland management in Qinghai‐Tibetan Plateau. PLoS ONE, 10, e0138969 https://doi.org/10.1371/journal.pone.0138969 26406891 10.1371/journal.pone.0138969 PMC4583544

[ece34103-bib-0099] Swets, J. A. (1988). Measuring the accuracy of diagnostic systems. Science, 240, 1285–1293. https://doi.org/10.1126/science.3287615 3287615 10.1126/science.3287615

[ece34103-bib-0100] Talbert, C. (2012). Software for assisted habitat modeling package for VisTrails (SAHM: VisTrails). Ver. 1. Fort Collins, CO: U.S. Geological Survey.

[ece34103-bib-0101] Tamburello, N. , Côté, I. M. , & Dulvy, N. K. (2015). Energy and the scaling of animal space use. American Naturalist, 186, 196–211. https://doi.org/10.1086/682070 10.1086/682070 26655149

[ece34103-bib-0102] Tateishi, R. , Zhu, L. , & Sato, H.P. (2000). The Land Cover Map for Central Asia for the YearGLC2000 database, European Commission Joint Research Centre, 2003. Retrieved from http://www-gem.jrc.it/glc2000.

[ece34103-bib-0103] Thomas, C. D. (2004). Extinction risk from climate change. Nature, 427, 145–148. https://doi.org/10.1038/nature02121 14712274 10.1038/nature02121

[ece34103-bib-0104] Thuiller, W. (2004). Patterns and uncertainties of species’ range shifts under climate change. Global Change Biology, 10, 2020–2027. https://doi.org/10.1111/j.1365-2486.2004.00859.x 10.1111/gcb.12727 PMC4340562 25200514

[ece34103-bib-0105] Turner, J. C. , Douglas, C. L. , Hallum, C. R. , Krausman, P. R. , & Ramey, R. R. (2004). Determination of critical habitat for the endangered Nelson's bighorn sheep in southern California. Wildlife Society Bulletin, 32, 427–448. https://doi.org/10.2193/0091-7648(2004)32[427:DOCHFT]2.0.CO;2

[ece34103-bib-0106] USGS (2016). U.S. Geological Survey Shuttle Radar Topography Mission (SRTM) resource archive. http://www.srtm.usgs.gov/index.php. Accessed 1 March 2017.

[ece34103-bib-0107] Valdez, R. , Michel, S. , Subbotin, A. , & Klich, D. (2016). Status and population structure of a hunted population of Marco Polo Argali Ovis ammon polii (Cetartiodactyla, Bovidae) in southeastern Tajikistan. Mammalia, 80, 49–57.

[ece34103-bib-0108] Valdez, R. , & Weinberg, P.J. (2011). Genus OVIS In Wilson D.E. & Mittermeier R.A. (Eds.), Handbook of the mammals of the world. Vol. 2. Hoofed mammals (pp. 726–739). Barcelona, Spain: Lynx Edicions.

[ece34103-bib-0109] Vuuren, D. P. , Edmonds, J. , & Kainuma, M. (2011). The representative concentration pathways: an overview. Climatic Change, 109, 5 https://doi.org/10.1007/s10584-011-0148-z

[ece34103-bib-0110] Warren, D. L. , & Seifert, S. N. (2011). Ecological niche modeling in Maxent: The importance of model complexity and the performance of model selection criteria. Ecological Applications, 21, 335–342. https://doi.org/10.1890/10-1171.1 21563566 10.1890/10-1171.1

[ece34103-bib-0111] Watanabe, M. , Suzuki, T. , O'ishi, R. , Komuro, Y. , Watanabe, S. , Emori, S. , & Takemura, T. (2010). Improved climate simulation by MIROC5: Mean States, Variability, and Climate Sensitivity. Journal of Climate, 23, 6312–6335. https://doi.org/10.1175/2010JCLI3679.1

[ece34103-bib-0501] Weaver, L. C. (2013). The potential for sustainable hunting management in the context of the Tajik National Park and the recently established Tajik World Heritage Site. http://www.wildlife-tajikistan.org/en/downloads/finish/2/69/0. Accessed 27 March 2018.

[ece34103-bib-0112] WHC (2013). Tajik National Park (Mountains of the Pamirs), World Heritage Committee, 37th session of the Committee (37 COM). Documents WHC‐13/37.COM/8B and WHC‐13/37.COM/INF.8B2, Phnom Penh, Cambodia, p. 49.

[ece34103-bib-0113] Wiens, J. A. , Seavy, N. E. , & Jongsomjit, D. (2011). Protected areas in climate space: What will the future bring? Biological Conservation, 144, 2119–2125. https://doi.org/10.1016/j.biocon.2011.05.002

[ece34103-bib-0114] Wilson, R. J. , Gutiérrez, D. , Gutierrez, J. , Martınez, D. , Agudo, R. , & Monserrat, V. J. (2005). Changes to the elevational limits and extent of species ranges associated with climate change. Ecology Letters, 8, 1138–1146. https://doi.org/10.1111/j.1461-0248.2005.00824.x 21352437 10.1111/j.1461-0248.2005.00824.x

